# Patterns of Missing Data With Ecological Momentary Assessment Among People Who Use Drugs: Feasibility Study Using Pilot Study Data

**DOI:** 10.2196/31421

**Published:** 2021-09-24

**Authors:** Kelly L Markowski, Jeffrey A Smith, G Robin Gauthier, Sela R Harcey

**Affiliations:** 1 Rural Drug Addiction Research Center University of Nebraska-Lincoln Lincoln, NE United States; 2 Department of Sociology University of Nebraska-Lincoln Lincoln, NE United States

**Keywords:** EMA, ecological momentary assessment, PWUD, people who use drugs, noncompliance, missing data, mobile phone

## Abstract

**Background:**

Ecological momentary assessment (EMA) is a set of research methods that capture events, feelings, and behaviors as they unfold in their *real-world* setting. Capturing data *in the moment* reduces important sources of measurement error but also generates challenges for noncompliance (ie, missing data). To date, EMA research has only examined the overall rates of noncompliance.

**Objective:**

In this study, we identify four types of noncompliance among people who use drugs and aim to examine the factors associated with the most common types.

**Methods:**

Data were obtained from a recent pilot study of 28 Nebraskan people who use drugs who answered EMA questions for 2 weeks. We examined questions that were not answered because they were *skipped*, they *expired*, the phone was switched *off*, or the *phone died* after receiving them.

**Results:**

We found that the phone being switched *off* and questions *expiring* comprised 93.34% (1739/1863 missing question-instances) of our missing data. Generalized structural equation model results show that participant-level factors, including age (relative risk ratio [RRR]=0.93; *P*=.005), gender (RRR=0.08; *P*=.006), homelessness (RRR=3.80; *P*=.04), personal device ownership (RRR=0.14; *P*=.008), and network size (RRR=0.57; *P*=.001), are important for predicting *off* missingness, whereas only question-level factors, including time of day (ie, morning compared with afternoon, RRR=0.55; *P*<.001) and day of week (ie, Tuesday-Saturday compared with Sunday, RRR=0.70, *P*=.02; RRR=0.64, *P*=.005; RRR=0.58, *P*=.001; RRR=0.55, *P*<.001; and RRR=0.66, *P*=.008, respectively) are important for predicting *expired* missingness. The week of study is important for both (ie, week 2 compared with week 1, RRR=1.21, *P*=.03, for *off* missingness and RRR=1.98, *P*<.001, for *expired* missingness).

**Conclusions:**

We suggest a three-pronged strategy to preempt missing EMA data with high-risk populations: first, provide additional resources for participants likely to experience phone charging problems (eg, people experiencing homelessness); second, ask questions when participants are not likely to experience competing demands (eg, morning); and third, incentivize continued compliance as the study progresses. Attending to these issues can help researchers ensure maximal data quality.

## Introduction

### Background

Ecological momentary assessment (EMA) is a collection of research methods used to study events, behaviors, and feelings as they unfold in their natural, *real-world* setting [1,2]. This is possible because participants are prompted to answer questions in real time, wherever they happen to be. Questions can be asked at specified times, randomly, or when certain events occur. Advanced technology, such as smartphones, facilitates this process by automating question prompts and time-stamping responses. Smartphones also often allow for the simultaneous collection of GPS location and Bluetooth proximity sensing, providing additional social context to EMAs and allowing questions to be prompted when participants are at or near certain locations or with or near specific others [3,4].

EMA provides many benefits for researchers, especially those studying vulnerable populations, such as people who use drugs (PWUD) [5]. For example, EMA facilitates the rapid collection of longitudinal data and, thereby, the study of causal relationships between precipitating factors and time-sensitive events such as relapse or the desire to use [1]. EMA also promotes more accurate reporting of sensitive behaviors such as substance use because multiple daily assessments reduce the time from behavior to recall and shorten the span of time to report on [6-9]. However, these advantages may be severely attenuated if participants do not respond to EMAs: high volumes of missing data threaten study validity and may lead to biased results and conclusions [1,5].

Motivated by validity concerns, a large body of literature examines EMA compliance or response rates to EMA questions. With respect to PWUD, a recent meta-analysis included 126 EMA-based studies [10]. Although the authors reported a wide range of compliance rates of 40%-100%, they concluded that EMA is largely feasible among this population, as 75% of all EMA prompts were answered on average [10]. Comparable rates have been reported among related populations, such as youth and adults who experience homelessness and who use drugs [11-13].

Although encouraging, these studies suggest that, on average, 25% of EMAs go unanswered among PWUD. Exploring the reason, past research has examined the effect of multiple predictors on noncompliance. Some studies find that demographic factors, such as age, gender, race or ethnicity, and education, influence noncompliance. Past work has found that older individuals, men, racial or ethnic minorities, and individuals with lower education respond to fewer EMAs [10,14-16]. Other studies have found that more mechanical factors related to study design are important, such as study duration, the daily number of EMAs, and EMA timing. Compliance tends to decrease with longer study periods, when more EMAs are asked per day, and when EMAs are asked in the morning [10,17-19].

This past work is informative, but it is limited in a major way: it does not distinguish between different *types* of missingness. For example, were the questions seen by the participants but deliberately skipped? Did participants fail to answer questions before they expired or *timed out*? Was the device switched off, meaning that the participants never received the question at all? Or perhaps did low battery force the phone to shut off, preventing participants from submitting answers?

It is crucial to distinguish between missing data types to identify specific barriers to providing data that participants face. Different patterns of missing data likely require different solutions to increase compliance. For example, the bulk of missingness in an individual’s EMA data may come from questions expiring. This may be because of participants having been unable or unwilling to answer questions while at work or with friends or family [10,20]. In this case, researchers may want to alter the design aspects of the EMA study itself, such as when questions are asked, to accommodate participants’ competing demands. On the other hand, missingness might primarily originate from battery dying or the device frequently being switched off. Here, participants may have experienced chronic issues with access to reliable charging, perhaps because of experiencing current homelessness [21]. Alternatively, participants may have been worried about confidentiality and data security related to GPS tracking, turning off their devices at or near certain locations [22,23]. When these latter issues are the most pressing, researchers may want to make sure to provide portable chargers for EMA devices and review data security protocols with participants before the study begins.

### Objective

In short, an EMA researcher should know which types of missing data are most likely to impact their study, as well as the factors associated with each missing data type. This would make it possible to more effectively plan a study to preempt noncompliance and strengthen validity. Toward this end, we used data from a recent pilot study with 28 PWUD in southeastern Nebraska to examine patterns in missing EMA data. First, we disaggregated four noncompliance types, including EMA questions that were not answered because they were skipped, they expired, the device was off, or the device died after receiving them. Then, we examine the factors associated with the two most prevalent types of missingness. We end the paper by offering targeted suggestions on how future EMA studies with PWUD can improve validity by reducing the two most common types of missing data.

## Methods

### Study Overview

The data were obtained from a 2-week pilot study, conducted in October 2020, that examined drug use in relation to daily interactions, social support, and well-being among PWUD. The study also tested the feasibility of using a smartphone-based EMA app, called the Open Dynamic Interaction Network (ODIN) [24,25], among this population. As our data were collected during the COVID-19 pandemic, extensive health and safety protocols were followed to maximize safety and minimize transmission risk [26].

### Participants and Recruitment

Participants were recruited from the Rural Health Cohort (RHC) study, a longitudinal data collection effort by the Rural Drug Addiction Research Center to study active drug users in rural Nebraska. Wave 1 of the RHC was collected in November 2019-March 2020 and consisted of 120 participants from southeastern Nebraska, recruited using respondent-driven sampling [27]. RHC participants were adults aged 19 years or older who used one or more illegal substances within 7 days of recruitment. We were given access to the names and phone numbers of 18 RHC participants who agreed to be contacted for participation in related studies and who satisfied our eligibility criteria: English-speaking adults who felt comfortable using a smartphone. Recruits often referred friends and other associates to our study, and we allowed these referrals (when eligible) to enroll. Our total enrollment included 28 PWUD—15 RHC participants and 13 referrals.

### Procedure

First, participants attended an intake appointment where, after consenting to participate, they completed an electronic survey including questions about demographic characteristics, drug use, social support, and daily interaction networks. Participants were given a smartphone (with the ODIN app installed), a phone charger, and a tutorial on the app and device, even if they had their own phone. Three different phone models were distributed: Nokia 2.3 (n=8), Motorola Moto E (n=11), and Motorola Moto E6 (n=9). Devices came with an unlimited talk, text, and data plan for the study period.

Second, the participants completed 2 weeks of EMA data collection. GPS location and Bluetooth proximity sensing were also collected (with consent) during this time. EMAs were sent through the ODIN app. All EMAs and display rules were stored locally on the phone via the app, meaning that neither Wi-Fi nor cell services were necessary for questions to be sent. All EMA data (as well as GPS and Bluetooth data) were stored on an encrypted database on the phone. Data uploads to a restricted access server were scheduled to occur every 20 minutes over a secure Sockets Layer connection and did require cell service (though any data not uploaded because of lack of access to service was archived until service was available).

EMA questions included momentary items (eg, those asking about the *right now* experience) and retrospective items (eg, those that asked about yesterday’s experience) [28]. Although most questions were prompted at specified times, some appeared at *random*, and there were two sets of event-contingent questions [29]. First, participants were asked to push a *button* (a feature within the ODIN app) any time they felt the desire to use drugs. Second, two items were prompted based on Bluetooth proximity to other study devices. Participants were asked a minimum of 104 questions each week. This number reflects the questions asked of all participants. Depending on participants’ responses to these questions, follow-up questions often ensued. Here, we focus only on the questions that all participants received, which excludes event-contingent items and follow-up questions.

Finally, participants attended an outtake appointment where they returned the study equipment and completed a second electronic survey plus a semistructured exit interview. The interview broadly asked about the participants’ experiences in the study [21].

### Compensation

Participants were compensated with up to US $120 in cash. At the end of the intake appointment, the participants were compensated with US $20. Participants were also compensated with US $20 at the end of the outtake appointment, where up to an additional US $20 was given as compensation for returning study equipment (US $5 for the charger and US $15 for the phone). The EMA portion of the study involved compensation as well (up to US $60), which was calculated weekly and was prorated on the number of questions answered (minimum of US $5 for 25 questions answered and maximum of US $30 per week for 88 questions answered or more). All compensation, including the EMA compensation schedule, was reviewed in detail with participants in the intake appointment as part of the consent procedure. The EMA compensation schedule was also outlined in the consent form, and each participant received a copy of the consent form in the intake appointment. Compensation earned for the EMA portion of the study was distributed at the end of the outtake appointment. Finally, participants were contacted via the study phone at the end of the first week to let them know how many questions they answered that week and how much compensation they earned.

### Measures

#### EMA Prompts

The EMA prompts were sent daily for 14 days. Each day, 13 questions were asked at three specified times. At 9 AM, three questions were asked about yesterday’s activities, hangout partners, and stressful interactions. At 12 PM, four questions were asked about yesterday’s drug use as well as needed, received, and given social support. At 7 PM, six questions were asked about current well-being and other psychosocial experiences. From Monday-Saturday only, two questions, sent between 2 PM and 5 PM, asked what participants were currently doing and feeling. On Sunday, only one question was asked at 4 PM about how frequently the participant desired to use drugs over the past week. As the same EMAs were asked each day at the same time, many participants came to expect them and incorporated them into their routine [21]. Each EMA took less than a minute to complete, meaning that participants spent less than 2 hours answering questions each week. EMA wording and other EMA characteristics have been provided in Table 1.

**Table 1:** Ecological momentary assessment questions and question characteristics.

Question Number	Question	Question type	Days asked	Time block asked
1	Which of the following activities did you do yesterday?	Select all that apply	Everyday	Morning
5	Who did you hang out with yesterday?	Select all that apply	Everyday	Morning
8	Thinking about your interactions from yesterday, how many of them were stressful?	Single-select	Everyday	Morning
13	Which of the following drugs did you use yesterday?^a^	Select all that apply	Everyday	Afternoon
20	Which of the following types of support did you need yesterday?	Select all that apply	Everyday	Afternoon
22	Which of the following types of support did you receive yesterday?	Select all that apply	Everyday	Afternoon
24	Which of the following types of support did you give to others yesterday?	Select all that apply	Everyday	Afternoon
26	What are you doing right now?	Text response	Monday-Saturday	Afternoon
27	How are you feeling right now?	Text response	Monday-Saturday	Afternoon
28	How depressed do you feel today?	Single-select	Everyday	Evening
29	How anxious do you feel today?	Single-select	Everyday	Evening
30	How lonely do you feel today?	Single-select	Everyday	Evening
31	There is no way I can solve some of the problems I have.	Single-select	Everyday	Evening
32	Today, it feels like people look down upon me because of my drug use.^a^	Single-select	Everyday	Evening
33	Today, it feels like people see me the way I want to be seen.	Single-select	Everyday	Evening
43	In the past week, how often did you want to use drugs other than alcohol, tobacco, and marijuana?^a^	Single-select	Sunday Only	Noon or Afternoon

^a^Question is a sensitive, drug-related question.

#### EMA Answers and Missingness

The participants had 2 hours to answer the EMAs. EMAs appeared on the phone as a notification on the home screen, which was accompanied by sound and phone vibrations. Although it was not possible for us to prevent participants from disabling these features, we requested that individuals not alter any phone settings. To access EMAs, participants could click the notification or click the ODIN app on the phone. Available questions were listed as buttons reading *Available Question* on the ODIN home screen. Once the questions were opened, participants could view the question and response options, and they could either click the *Submit* button to progress to the next question or hit the *Back* button to exit the question.

If participants opened the EMA question, provided an answer, and clicked the *Submit* button before the 2 hours elapsed, we coded that question as *Answered*. However, if EMA questions were not answered before the 2 hours elapsed, those questions were broadly considered *Missing*. The ODIN app records four distinct types of missingness, which depend on two key factors: clicking the *Submit* button and the phone being switched on.

First, if the participant opened an available EMA question and clicked the *Submit* button without providing an answer, participants effectively skipped the question as in traditional surveys. In these instances, the question was recorded as *Skipped*. The important point here is that the participant opened the EMA and made a deliberate choice to provide no answer to the question.

Second, if the participant did not provide an answer or skip past an available EMA question but the phone was on as the 2-hour time frame elapsed, these questions were recorded as *Expired*. A question may be *Expired* if the EMA was never opened or if participants opened the EMA but clicked the *Back* button to exit it. Thus, to be *Expired*, the phone must be on, and there must be no response submitted (ie, no answer and not deliberately skipped).

Third, if an EMA question was available but the phone battery died and the phone stayed switched off as the 2-hour time frame elapsed, questions were recorded as *Phone Died*. Crucially, these questions were not *Answered* or *Skipped*, *and* participants did not turn the phone back on within the 2-hour time frame. If participants did turn the phone back on before the time frame elapsed, participants were still able to provide an answer (meaning that questions could also instead be *Skipped* or *Expired* as outlined above).

Finally, if a question was not recorded as any of the previous options, this means the phone was switched off from the time the questions were made available until after the 2-hour time frame elapsed. We manually coded these instances as *Phone Off*. Importantly, if participants turned the phone on before the time frame elapsed, questions were made available, and participants were still able to provide an answer in the remaining time frame (meaning that questions could also instead be *Skipped* or *Expired* as outlined above).

As a final point of consideration, the ODIN app and the specific survey schedule used here were extensively beta-tested by the authors before data were collected. This process included using the same phone models as those distributed to participants, replicating the sources of missingness outlined above, and verifying the resulting missingness codes as beta-testing ensued. This, combined with the rigorous testing and validation completed by the ODIN development team, means that we can be confident in the data collected and presented here.

#### Participant and Social Network Characteristics

Demographic and social network information was collected from participants in the intake survey. Participants indicated how old they were in years. For gender, they indicated if they were *Man/male* or *Woman/female* (1 participant was trans woman or male-to-female transgender person; 0=man/male; 1=woman/female). For race, participants selected all that applied from the following list: *White, Black or African American*, *American Indian or Alaska Native, Asian, Native Hawaiian or Pacific Islander,* and *some other race*. Participants also indicated whether they were Hispanic or Latino (yes or no). A racial and ethnic minority indicator was created from these race and ethnicity questions (0=non-Hispanic/Latino White; 1=Black, Hispanic/Latino, and Multiracial). For homelessness, participants indicated if they were currently homeless (0=no; 1=yes). For education, participants selected the highest level of education they completed from the following list: *Less than high school, Completed high school or GED, Some college, Completed 2-year degree, Completed 4-year degree, and Graduate or professional degree*. For income, participants indicated their total household income (in US $) in the last 12 months. Categories started at *Less than $5000*, then they ranged from *$5001 to $10,000* to *More than $100,000,* increasing in increments of US $10,000. To summarize this variable, we replaced each category with the midpoint of its range in dollars. Participants selected their current employment status from the following list: *Employed full-time, Employed part-time, A homemaker, A full-time student, Retired, Disability–temporary, Disability–permanent, Unemployed,* and *Other*. We combined temporary and permanent disability into a *Disabled* category and merged *Student* and *Retired* into the *Other* category. In the exit interview, participants were asked if they had their own personal cell phones (0=no; 1=yes). The final two following items were obtained from the intake survey. Participants listed the initials of up to 10 people they interacted with most on a regular basis. From this, we summed the number of people listed to create a network size variable. Finally, participants indicated the substances that they used either alone or with each person in their network within the past month. The list included *Marijuana, Methamphetamine, Amphetamine, Cocaine, Heroin, Prescription opioids*, and *Something else.*

### Analytic Strategy

We began by examining compliance and noncompliance rates. Then, we examined how a variety of factors are generally associated with missingness before exploring the relationship between these factors and the two most prevalent types of missingness. To do this, we estimate 2 generalized structural equation models. The first included a binary outcome variable capturing all noncompliance (any *Missingness*) relative to compliance (*Answered*). The second included a multinomial outcome variable capturing the type of noncompliance (*Off* vs *Expired*) relative to compliance (*Answered*). Observations were at the question level and were clustered within a latent person-identifying variable to account for within-person dependencies in the data. Missing predictor values were either imputed at the mean (eg, for network size) or were manually entered based on information from face-to-face interactions during in-person appointments. Certain variables, including education, income, employment, and substance use, were highly correlated with other variables (eg, homelessness status). We excluded these variables from both models because including them would have caused problems with estimation. Stata 15 (StataCorp LLC) was used to estimate the model. Significance was interpreted using the conventional .05, .01, and .001 levels.

## Results

### Sample Characteristics

Table 2 shows characteristics of the sample. The average age was about 41 years old (SD 14.97; range 22-70). About one-fifth of the sample were women (6/28, 21% of participants). Just over one-third (10/28, 36% of participants) identified as people of color. Exactly half (14/28, 50% of participants) were currently experiencing homelessness. The participants were largely highly educated as over two thirds (18/28, 64% of participants) had some college education or more. Average income was US $13,981 (income ranged from US $2500-$95,000). Just over one-third of the participants were employed part-time or full-time (10/28, 36% of participants) and just over one-third was unemployed (10/28, 36% of participants) Just under three-fourths (20/28, 71% of participants) had their own personal cell phone. Participants listed an average of about 7 daily interaction partners (network size ranged from 1-10). Marijuana was the most reported substance used by participants in the last month (18/28, 64% of participants), followed by methamphetamine (11/28, 39% of participants), cocaine (5/28, 18% of participants), and prescription opioids (5/28, 18% of participants).

**Table 2:** Demographic descriptive statistics (N=28).

Demographic characteristics		Value
Age (years), mean (SD)		40.85 (14.97)
Women, n (%)		6 (21)
Racial and ethnic minority, n (%)		10 (36)
Currently homeless, n (%)		14 (50)
**Education, n (%)**		
	Less than high school	3 (11)
	High school	7 (25)
	Some college	8 (29)
	Completed 2-year degree	7 (25)
	Completed 4-year degree	3 (11)
Income (US $), mean (SD)		13981.48 (18928.75)
**Employment, n (%)**		
	Full-time	6 (21)
	Part-time	4 (14)
	Disabled	5 (18)
	Unemployed	10 (36)
	Other	3 (11)
Personal device, n (%)		20 (71)
Network size, mean (SD)		7.15 (2.47)
**Substance use, n (%)**		
	Marijuana	18 (64)
	Methamphetamine	11 (39)
	Amphetamine	2 (7)
	Cocaine	5 (18)
	Prescription Opioids	5 (18)
	Other	3 (11)

### Sample Participation

Of the 28 participants in our sample, 22 (79%) completed 2 weeks of data collection, 3 (11%) ended their participation 1 day early, providing data for only 13 out of 14 days. Furthermore, 7% (2/28) participants lost or damaged their study phone during the study; as a result, neither could provide data while coordinating a phone replacement and thus only provided data for 11 days. One had to drop out of the study and only provided data for 9 days. In all analyses, we adjust for these *missing* days by limiting the days under consideration to those where participants had a working study phone in their possession.

### Compliance and Missingness

Table 3 shows compliance rates across all question-instances. Out of 5615 questions, participants provided responses to 3752, overall compliance rate of about 66.82%. Compliance is slightly higher in the first week compared with the second (2021/2867, 70.49% vs 1731/2748, 62.99%), a difference that is statistically significant (*Χ*^2^_1_=35.6; *P*<.001). Across days of the week, Sunday has the lowest compliance rate (496/770, 64.42%), whereas Tuesday has the highest (558/810, 68.89%). Neither this nor any other day of week comparisons are significantly different.

**Table 3:** Compliance and noncompliance rates (question-instances; N=5615).

	Values, n (%)						
	Answered	Missing (all)	Missing (off)	Missing (expired)	Missing (phone died)	Missing (skipped)	Total
Full study	3752 (66.82)	1863 (33.18)	916 (16.31)	823 (14.66)	101 (1.8)	23 (0.41)	5615 (100)
Week 1	2021 (70.49)	846 (29.51)	476 (16.6)	324 (11.3)	35 (1.22)	11 (0.38)	2867 (100)
Week 2	1731 (62.99)	1017 (37.01)	440 (16.01)	499 (18.16)	66 (2.4)	12 (0.44)	2748 (100)
Sunday	496 (64.42)	274 (35.58)	120 (15.58)	143 (18.57)	8 (1.04)	3 (0.39)	770 (100)
Monday	548 (66.42)	277 (33.58)	138 (16.73)	127 (15.39)	10 (1.21)	2 (0.24)	825 (100)
Tuesday	558 (68.89)	252 (31.11)	116 (14.32)	128 (15.8)	7 (0.86)	1 (0.12)	810 (100)
Wednesday	534 (67.17)	261 (32.83)	121 (15.22)	110 (13.84)	27 (3.4)	3 (0.38)	795 (100)
Thursday	536 (67.42)	259 (32.58)	137 (17.23)	104 (13.08)	13 (1.64)	5 (0.63)	795 (100)
Friday	526 (66.16)	269 (33.84)	156 (19.62)	93 (11.7)	12 (1.51)	8 (1.01)	795 (100)
Saturday	554 (67.15)	271 (32.85)	128 (15.52)	118 (14.3)	24 (2.91)	1 (0.12)	825 (100)

**Figure 1:**
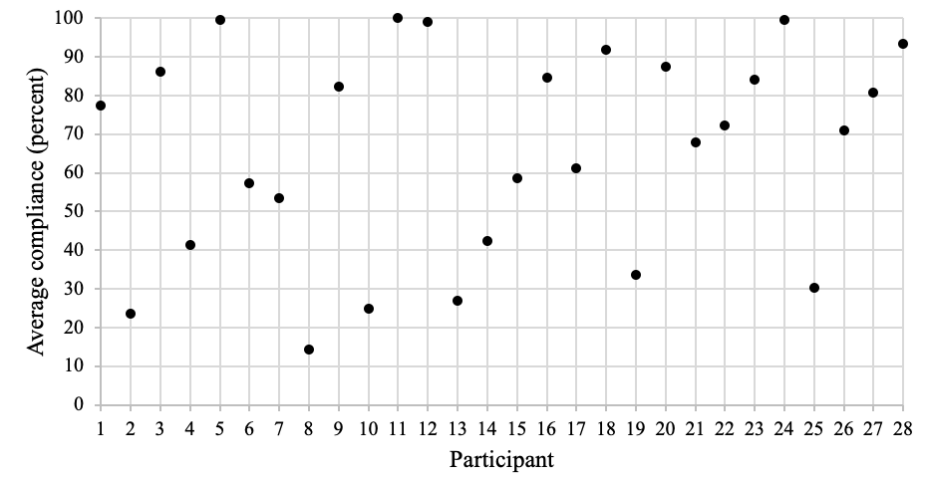
Average compliance rate across participants.

When examining compliance rates across participants (Figure 1), compliance rates vary widely across the sample. Approximately 43% (12/28 participants) fell below the average compliance rate of 66.82% (3752/5615). Furthermore, 1 participant had the lowest overall compliance rate (participant 8: 30/208, 14.4%), and 4 participants had compliance rates of 99% or higher (participant 5: 207/208, 99.5%; participant 11: 208/208, 100%; participant 12: 206/208, 99%; participant 24: 207/208, 99.5%), missing two questions or less across the full study period.

Table 3 also shows the noncompliance rates by the missingness type. Data were primarily missing (1863/5615, 33.18% total) because the phone was *Off* (916/5615, 16.31%), followed by *Expired* questions (823/5615, 14.66%), a difference that was statistically significant (*Χ*^2^_1_=5.84; *P*<.02). Moreover, 1.8% (101/5615) of questions were missing because the *Phone Died* and 0.41% (23/5615) were missing because participants *Skipped* the question. Although the rates of *Off* noncompliance appear relatively consistent across weeks, *Expired* noncompliance significantly increased in week 2 (499/2748, 18.16%) compared with week 1 (324/2867, 11.3%) (*Χ*^2^_1_=52.78; *P*<.001). Across days of the week, *Off* noncompliance is highest on Friday (156/795, 19.6%). *Expired* noncompliance is highest on Sunday (143/770, 18.6%).

### Predictors of Missingness

Table 4 presents logistic regression model predicting any missingness. The results ignore the specific type of missing data and simply predict if the question was answered or not (0=answered; 1=not answered). Results are presented as odds ratios. We can interpret these effects in terms of the odds of missingness. Scores below 1 indicate decreased odds of missingness; scores above 1 indicate increased odds of missingness.

In this model, we find very few significant effects that predict missingness. Only 1 day of the week, week of study, and gender were significant. Compared with Sunday, the odds of missingness decreased on Tuesdays (OR 0.75; *P*=.02). Odds of missingness increased in week 2 relative to week 1 (OR 1.55; *P*<.001). Odds of missingness were lower for women than for men (OR 0.15; *P*=.02). Supplemental analyses predicting above-average compliance at the day level across participants yielded similar results (Multimedia Appendix 1).

**Table 4:** Multilevel logistic model results predicting noncompliance (N=5615)^a^.

Predictor			Missing, odds ratio (95% CI)
**Question level**			
	Morning	0.89 (0.73-1.07)	
	Evening	0.86 (0.74-1.01)	
	Monday	0.85 (0.66-1.10)	
	Tuesday	0.75^b^ (0.58-0.97)	
	Wednesday	0.82 (0.64-1.06)	
	Thursday	0.78 (0.61-1.01)	
	Friday	0.88 (0.68-1.13)	
	Saturday	0.81 (0.63-1.05)	
	Week 2	1.55^c^ (1.35-1.78)	
	Sensitive drug question	0.90 (0.74-1.10)	
**Participant level**			
	Moto E	0.27 (0.06-1.26)	
	Moto E6	0.37 (0.08-1.82)	
	Personal device	0.35 (0.09-1.42)	
	Age	0.96 (0.91-1.01)	
	Women	0.15^b^ (0.03-0.80)	
	Homeless	2.15 (0.62-7.47)	
	Racial and ethnic minority	0.60 (0.16-2.21)	
	Network size	0.77 (0.56-1.03)	

^a^The reference category for morning and evening was afternoon. The reference category for Monday-Saturday was Sunday. The reference category for Moto E and Moto E6 was Nokia 2.3. The unstandardized coefficient for the latent, person-identifying variable accounting for within-person dependencies in the data is 2.25 (95% CI 1.24-4.09).

^b^*P*<.05.

^c^*P*<.001.

### Predictors of Expired and Off Missingness

How do the same factors relate to different types of missingness? Table 5 presents the model results predicting *Expired* and *Off* missingness—the two most prevalent types of missingness in our data. The left-side of the table presents the relative risk ratio estimates for *Expired* question missingness compared with questions *Answered*; the right-side presents relative ratio risk estimates for *Off* missingness compared with questions *Answered*. As with Table 4, we can interpret these effects in terms of factor changes in the odds of missingness, although here we have separate estimates for each type of missing data.

**Table 5:** Multilevel multinomial logistic model results predicting specific noncompliance (N=5491)^a^.

Predictor		RRR^b^ (95% CI)	
		Expired	Off
**Question level**			
	Morning	0.55^c^ (0.43-0.72)	1.18 (0.93-1.49)
	Evening	0.90 (0.75-1.09)	0.76^d^ (0.63-0.93)
	Monday	0.74 (0.54-1.01)	1.00 (0.72-1.39)
	Tuesday	0.70^e^ (0.51-0.95)	0.80 (0.57-1.12)
	Wednesday	0.64^d^ (0.47-0.87)	0.93 (0.66-1.30)
	Thursday	0.58^c^ (0.42-0.80)	1.02 (0.73-1.41)
	Friday	0.55^c^ (0.40-0.77)	1.23 (0.88-1.70)
	Saturday	0.66^d^ (0.48-0.90)	0.91 (0.66-1.27)
	Week 2	1.98^c^ (1.67-2.35)	1.21^e^ (1.02-1.45)
	Sensitive drug question	0.89 (0.70-1.13)	0.94 (0.73-1.21)
**Participant level**			
	Moto E	0.32 (0.07-1.58)	0.20^e^ (0.04-0.96)
	Moto E6	0.32 (0.06-1.65)	0.62 (0.12-3.34)
	Personal device	0.90 (0.21-3.81)	0.14^d^ (0.03-0.59)
	Age	0.97 (0.92-1.02)	0.93^d^ (0.88-0.98)
	Women	0.20 (0.03-1.17)	0.08^d^ (0.01-0.48)
	Homeless	1.36 (0.37-4.92)	3.80^e^ (1.04-13.81)
	People of color	0.34 (0.09-1.30)	1.70 (0.44-6.58)
	Network size	0.94 (0.69-1.29)	0.57^c^ (0.42-0.78)

^a^The reference category for morning and evening was afternoon. The reference category for Monday-Saturday was Sunday. The reference category for Moto E and Moto E6 was Nokia 2.3. The unstandardized coefficient for the latent, person-identifying variable accounting for within-person dependencies in the data is 2.39 (95% CI 1.32-4.34).

^b^RRR: relative risk ratio.

^c^*P*<.001.

^d^*P*<.01.

^e^*P*<.05.

Table 5 offers a clearer picture than our previous results, suggesting that missing data do, in fact, need to be differentiated by type. Beginning with *Expired* missingness, only question-level predictors are significant, including day of the week, week of study, and time of day. The risk of expired missingness decreased when questions were asked in the morning compared with afternoon (relative risk ratio [RRR]=0.55; *P*<.001). Compared with Sunday, the risk of expired missingness decreased on Tuesday (RRR=0.70; *P*=.02), Wednesday (RRR=0.64; *P*=.005), Thursday (RRR=0.58; *P*=.001), Friday (RRR=0.55; *P*<.001), and Saturday (RRR=0.66; *P*=.008). In addition, the risk of expired missingness increased in week 2 relative to week 1 (RRR=1.98; *P*<.001). Note that we do not see significant differences in question sensitivity or by any participant-level predictor.

In contrast, nearly all participant-level predictors are significant for *Off* missingness, plus a few question-level predictors. Having a personal device, age, gender, homelessness, and network size show significant coefficients predicting *Off* missingness. In all but one case, these predictors make *Off* missingness less likely. The risk of *Off* missingness decreases for those with a personal device (RRR=0.14; *P*=.008) and for women (RRR=0.08; *P*=.006) as well as decreases with each additional year of age (RRR=0.93; *P*=.005) and each additional network member (RRR=0.57; *P*=.001). Phone type is also significant, as *Off* missingness decreases with the Moto E phone model compared with the Nokia 2.3 (RRR=0.20; *P*=.045). On the other hand, *Off* missingness increases for those who are currently homeless (RRR=3.80; *P*=.04). We observe no significant effect for people of color. Finally, the risk of *Off* missingness decreases when questions are asked in the evening compared with noon or afternoon (RRR=0.76; *P*=.007) but again increases in week 2 compared with week 1 (RRR=1.21; *P*=.03).

## Discussion

### Principal Findings

In this study, we examined patterns in EMA missingness using data from a pilot study on 28 PWUD. Building upon previous work on EMA compliance or noncompliance, our study uniquely focused on the *type* of noncompliance. We differentiated between missed questions where the phone was switched off from those where the question expired, was skipped, or was not answered because the phone battery died. This is important because different types of noncompliance signal different issues participants may face in EMA studies, which in turn require unique solutions to increase compliance.

Our results yielded several interesting and important findings. First, our results suggest that noncompliance is primarily attributable to either the phone being switched off or questions expiring, as these comprised about 93.34% (1739/1863) of our missing questions and were almost equally prevalent. Thus, *Off* and *Expired* missingness are likely the most important missing data problems that EMA researchers face. In contrast, *Phone Died* and *Skipped* missingness comprised only about 6.65% (124/1863) of our missing questions and were relatively rare occurrences. This suggests that participants are not likely to skip many questions and that phones tend to remain dead for extended periods (thus becoming *Off* missingness).

Second, our results highlight the importance of separating missingness by type. We found few consistent effects in the model predicting any kind of missingness, although a clear story emerged when we disaggregated missingness into *Expired* and *Off* categories. Although past work has found that men and older individuals miss more EMAs in general [14-16], we found that these differences were only in relation to *Off* missingness: women (similar to past work) and older individuals (different from past work) missed fewer questions because the phone was switched off. This provides mixed evidence for demographic differences documented by other work; importantly, it also suggests that any demographic differences seen in past work may be solely because of participants having the phone switched off, as we found that younger individuals and men were not more likely to miss questions via expiration once received by the phone.

In contrast, we also found question-level differences, though the bulk of them were only in relation to *Expired* missingness. Although previous work has found that more questions are missed in the morning [17], we found that questions were less likely to expire in the morning. Despite the difference, this suggests that questions that expire may be less about the individual and more about mechanical study design features. We did find one major exception to this trend: week of study was relevant for noncompliance in general, as the second week predicted both *Expired* and *Off* missingness [18,19].

Another important aspect of our results is that we examined additional factors that have not been widely included in past EMA studies on compliance. That is, we assessed the effect of being currently homeless, network size, and having a personal device on EMA missingness. We found significant findings with relation to each: consistent with the idea that demographic differences relate to *Off* missingness, these variables significantly predicted only this type, with homelessness increasing missingness, whereas each additional network member and having a personal device decreased missingness. Assessing these variables in EMA studies is likely important because they may signal structural factors that are important for predicting EMA compliance, such as access to resources and time spent on mobile devices [30]. On a final note, we found that, compared with the Nokia 2.3 phone model, Moto E predicted less *Off* missingness. Although these devices were very similar in terms of size and features, many participants informally mentioned to research staff that the Nokia phone was less enjoyable to use and navigate. This may help explain the finding while also serving as an important reminder for researchers when selecting phone models for use in their studies.

### Implications for Future Studies Using EMA

Overall, these results have important implications for researchers planning EMA studies with at-risk, vulnerable populations. Below, we suggest a three-pronged strategy for how researchers can minimize noncompliance. We differentiate between factors that are (1) specific to having the phone off, (2) specific to the questions expiring, and (3) common to both types of missingness.

First, researchers should attempt to minimize factors that make it more likely for participants to have the phone off, most clearly linked here to individual-level attributes. For instance, homeless individuals (though also likely individuals from other at-risk, low socioeconomic status populations) may have limited access to reliable charging even if a phone charger is provided to participants. In this case, chronically experiencing low phone battery may be an issue influencing long-term *Phone Off* missingness. In this case, it is crucial to make the phones easily chargeable beyond simply providing a phone charger (if the phone is study provided). Researchers could provide portable chargers as well as a list of locations where phones can be charged safely and free [31]. It would also be wise to screen for any clear patterns of having the phone off while the study is underway, especially as it may relate to characteristics such as network size and phone model. For example, a researcher could check time-stamped GPS information to identify which participants have the phone off during key periods of the day and contact them as necessary to remind them about the study.

Second, researchers should attempt to minimize the factors that lead to question expiration. Here, the main concern is when the questions are prompted, especially the time of day (questions were less likely to be expired in the morning than in the afternoon) but also the day of the week (questions were less likely to expire on all other days, except Monday, compared with Sunday). This suggests minimizing questions asked during the end of the weekend and beginning of the week, if possible, and concentrating questions in the morning when EMAs may be more readily incorporated as part of a daily routine (eg, part of getting ready before work). The results also suggest offering longer than 2 hours to answer the questions or allowing participants to *suspend* EMAs that take place during a period of time when participants may be temporarily unavailable (eg, while driving, in a meeting) [32]. Researchers should balance these options with the types of EMAs asked. For example, retrospective items asking about past experiences may be better candidates for *suspension* than momentary items that are sensitive to timing because they ask about the *right now* experience [28].

Finally, a researcher should pay close attention to the participants dropping off in the second (or subsequent) weeks of the study. Fixing this problem has the potential to yield particularly large returns, as this is one of the only factors common to both *Off* and *Expired* missingness. A researcher could attempt to limit participant fatigue or boredom with the study by creating a more nuanced compensation structure, such that the amount participants earn increases as the study proceeds. It may also be useful to have participants come in midstudy for a re-engagement *check-in* to distribute to-date compensation and remind them of potential future compensation. In our case, we contacted participants midway through the study (eg, at the end of the first week) to alert them of the number of questions answered and compensation earned. More involved interactions were not possible because COVID-19 protocols limited the number of face-to-face interactions between participants and field staff [26]. We expect that more extensive check-ins and compensation opportunities may reinvigorate study commitment and engagement [33].

### Limitations

There are a few limitations to this study. First, our sample may not be representative of PWUD in general. It would be useful for future work to examine the rates of different types of missingness in EMA studies with larger samples of PWUD. Second, the extent to which participants turned off phones because of concerns about GPS tracking is unclear [22,23]. Although this topic did not emerge in the exit interviews with participants [21], it was also not a specific point of emphasis in the interview protocols. Future research should ask participants more targeted questions about motivations and instances of the phone deliberately being turned off so that additional actions can be taken to preempt this kind of noncompliance. Last, there are several other participant characteristics that may have uniquely contributed to either kind of missingness. For example, although some studies note that substance use frequency may impact participants’ ability or willingness to consistently complete EMA prompts [5,10,34], we could not assess this because use was highly correlated with other key predictors in the model, which would have caused problems with the estimates. Education and income exhibited similar issues. Future work should examine these and other potential factors, such as substance use disorder diagnosis, on larger samples with greater demographic variability.

Despite these limitations, our study reveals novel and important information about noncompliance in EMA studies with PWUD. As we were uniquely able to disaggregate different types of noncompliance, we showed which types are (and are not) likely to pose problems for researchers, which can inform planning for future EMA studies. By anticipating likely sources of missing data and preemptively enacting solutions to address it, research can work to ensure maximal compliance so that the advantages of EMA can be retained.

## Appendix

Multimedia Appendix 1 Multilevel model results predicting the average daily compliance rate (N=384).

## Abbreviations

EMA: ecological momentary assessment
ODIN: Open Dynamic Interaction Network
PWUD: people who use drugs
RHC: Rural Health Cohort
RRR: relative risk ratio
