# Supplementary material for: Patterns of Missing Data With Ecological Momentary Assessment Among People Who Use Drugs: Feasibility Study Using Pilot Study Data
Source: JMIR Form Res. 2021 Sep 24;5(9):e31421. doi: 10.2196/31421 (PMC8501406; doi:10.2196/31421)
Supplement: Multimedia Appendix 1 [file formative_v5i9e31421_app1.docx]

In this Appendix, we present model results predicting compliance (instead of non-compliance). Importantly, compliance is assessed in terms of questions answered each day. To predict above/below average daily compliance, we aggregated the number of questions participants answered each day (meaning that each participant has 14 or less datapoints on the dependent variable) and centered these values on the study’s overall average compliance rate (66.82%, which is about 10 questions per day Monday-Saturday and 9 questions per day on Sunday). The results can be interpreted in terms of how the number of questions answered increases above or decreases below the average number of questions answered as a factor of each variable. Overall, these results are nearly identical to those presented in Table 3: daily compliance drops below average in week two compared to week one, increases above average for women compared to men, and increases above average with each additional network member listed.

| Predictor | Missing | |
| --- | --- | --- |
|  | OR | CI |
|  |  |  |
| ***Question-Level*** |  |  |
|  |  |  |
| **Monday** |  |  |
|  | -0.05 | -1.47, 1.36 |
| **Tuesday** |  |  |
|  | 0.13 | -1.29, 1.54 |
| **Wednesday** |  |  |
|  | -0.31 | -1.72, 1.10 |
| **Thursday** |  |  |
|  | -0.21 | -1.62, 1.21 |
| **Friday** |  |  |
|  | -0.33 | -1.75, 1.10 |
| **Saturday** |  |  |
|  | 0.05 | -1.36, 1.47 |
| **Week two** |  |  |
|  | -1.09** | -1.84, -0.33 |
| ***Participant-Level*** |  |  |
|  |  |  |
| **Moto E** |  |  |
|  | 2.33 | -0.66, 5.32 |
| **Moto E6** |  |  |
|  | 1.18 | -1.93, 4.29 |
| **Personal device** |  |  |
|  | 1.84 | -0.90, 4.58 |
| **Age** |  |  |
|  | 0.09 | 0.00, 1.19 |
| **Women** |  |  |
|  | 3.86* | 0.59, 7.13 |
| **Homeless** |  |  |
|  | -1.83 | -4.25, 0.59 |
| **Non-white** |  |  |
|  | 0.95 | -1.59, 3.49 |
| **Network size** |  |  |
|  | 0.76** | 0.18, 1.24 |

Notes: * = *P<.05* ** = *P<.01* *** = *P<.001.* The reference category for Monday-Saturday is Sunday. The reference category for Moto E and Moto E6 is Nokia 2.3. The unstandardized coefficient for the latent, person-identifying variable accounting for within-person dependencies in the data is 7.82 (CI = 4.33, 14.16).
